# Supplementary figures and images for: The scaffolding protein flot2 promotes cytoneme-based transport of wnt3 in gastric cancer
Source: eLife. 2022 Aug 30;11:e77376. doi: 10.7554/eLife.77376 (PMC9457691; doi:10.7554/eLife.77376)

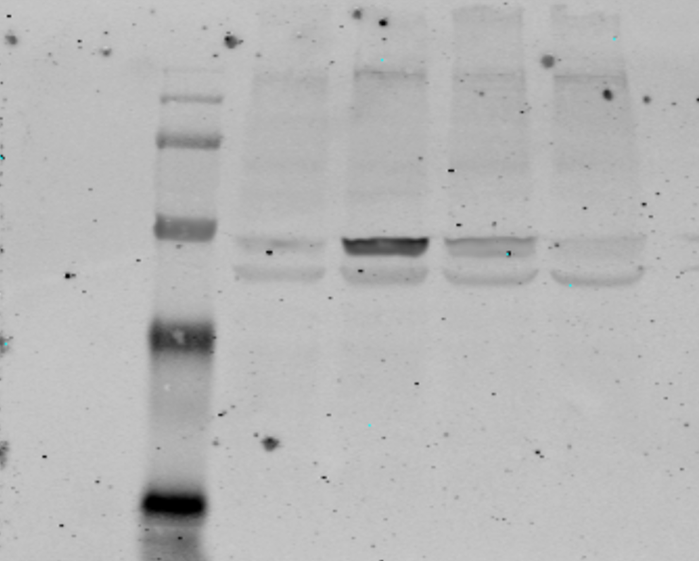

Supplement: Figure 3—source data 2. — Beta-actin was used as the loading control. [file elife-77376-fig3-data2.zip › Figure3-Source Data2/Figure 3A - Source Data 1.png]

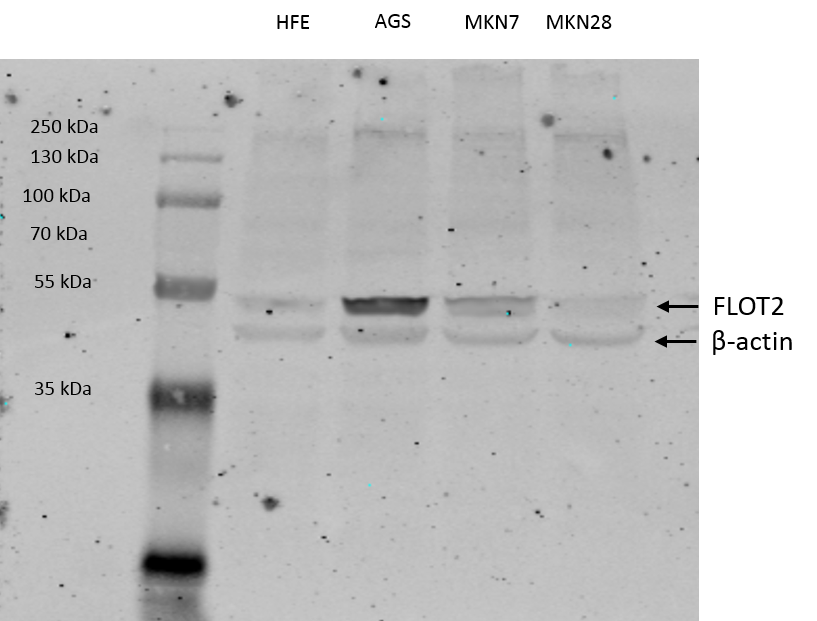

Supplement: Figure 3—source data 2. — Beta-actin was used as the loading control. [file elife-77376-fig3-data2.zip › Figure3-Source Data2/Figure 3A - Source Data 1b.png]

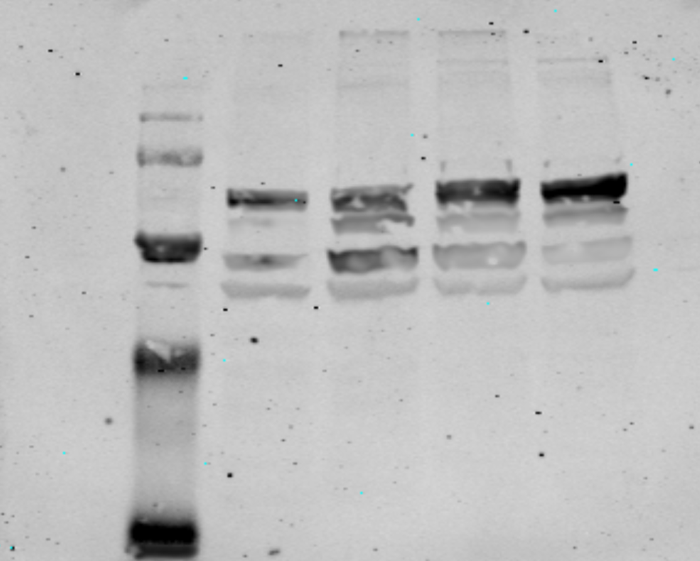

Supplement: Figure 3—source data 2. — Beta-actin was used as the loading control. [file elife-77376-fig3-data2.zip › Figure3-Source Data2/Figure 3A - Source Data 2.png]

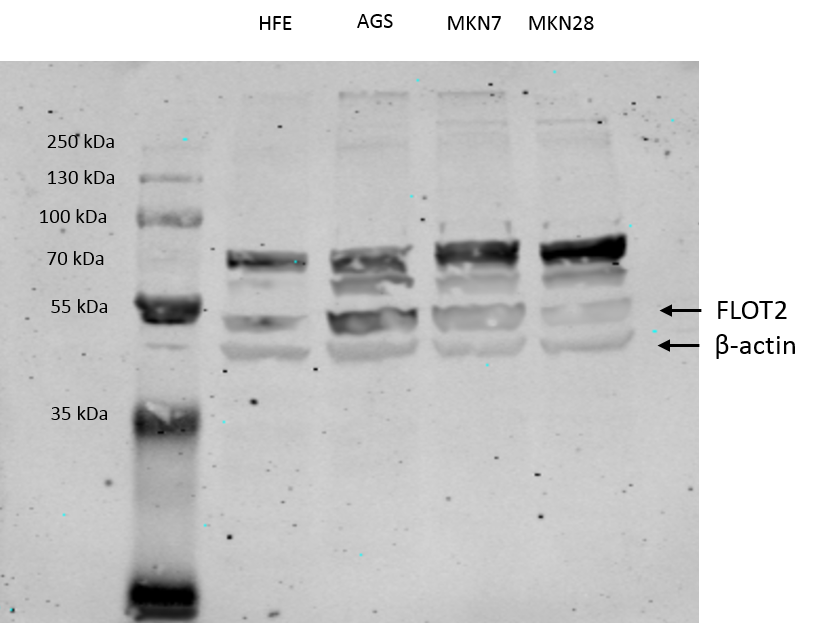

Supplement: Figure 3—source data 2. — Beta-actin was used as the loading control. [file elife-77376-fig3-data2.zip › Figure3-Source Data2/Figure 3A - Source Data 2b.png]

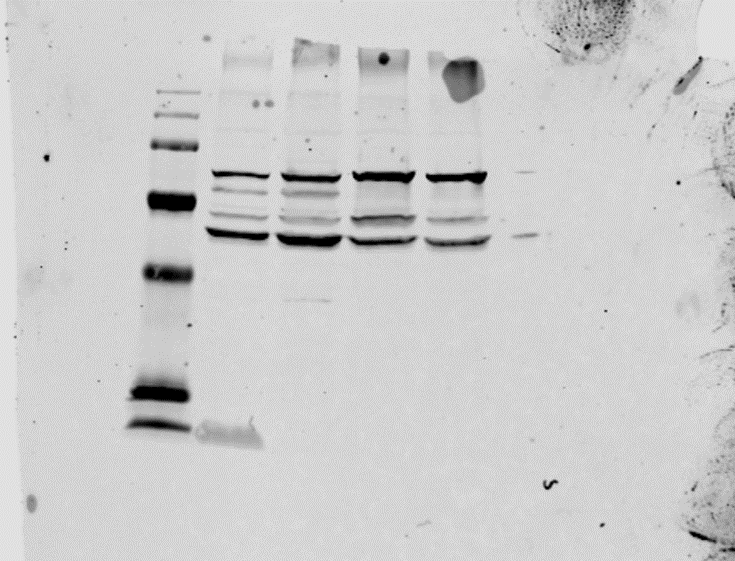

Supplement: Figure 3—source data 2. — Beta-actin was used as the loading control. [file elife-77376-fig3-data2.zip › Figure3-Source Data2/Figure 3A - Source Data 3.png]

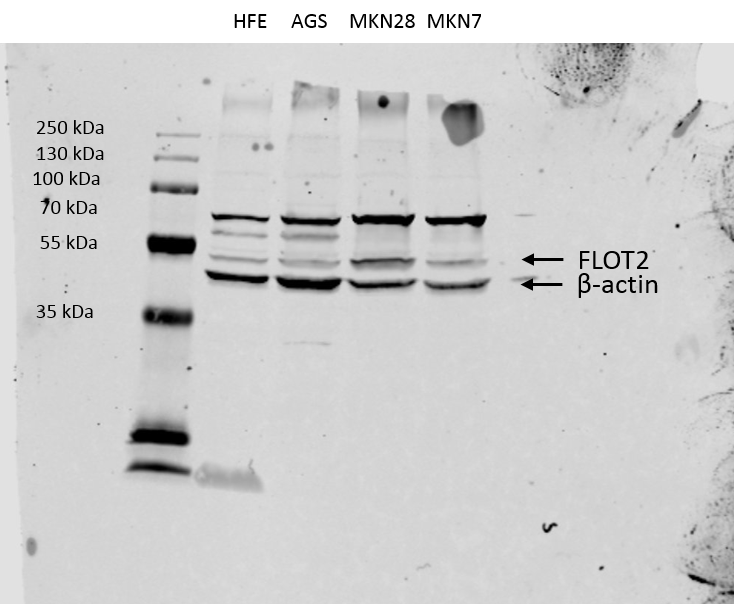

Supplement: Figure 3—source data 2. — Beta-actin was used as the loading control. [file elife-77376-fig3-data2.zip › Figure3-Source Data2/Figure 3A - Source Data 3b.png]

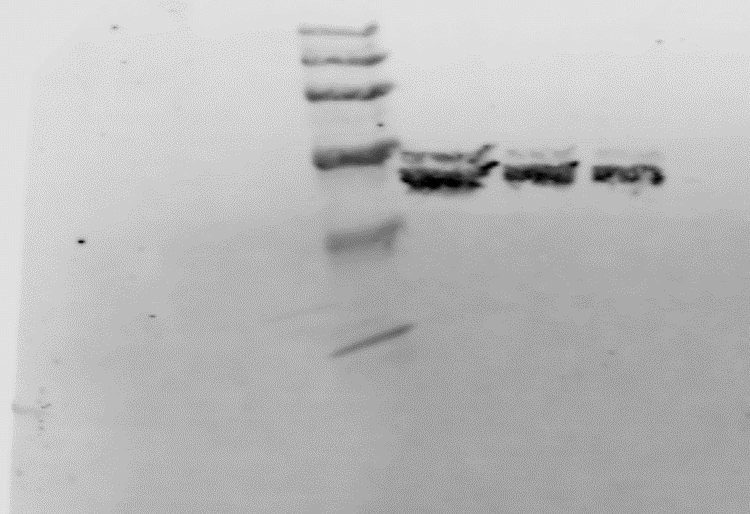

Supplement: Figure 3—figure supplement 1—source data 2. [file elife-77376-fig3-figsupp1-data2.zip › Figure3-supplement figure1-Source Data/Figure3-supplement figure1D-Source Data 1.png]

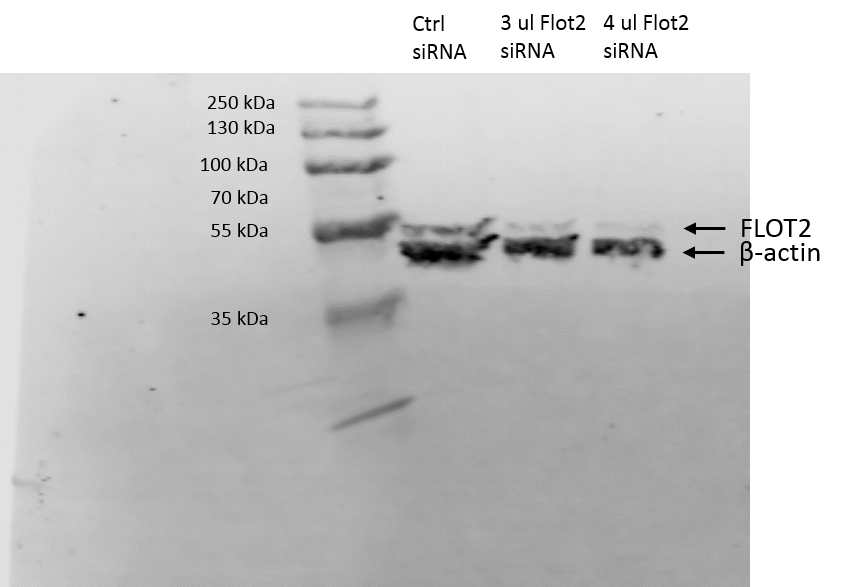

Supplement: Figure 3—figure supplement 1—source data 2. [file elife-77376-fig3-figsupp1-data2.zip › Figure3-supplement figure1-Source Data/Figure3-supplement figure1D-Source Data 1b.png]
